# The importance of the nuclear positioning of the *PPARG* gene for its expression during porcine in vitro adipogenesis

**DOI:** 10.1007/s10577-019-09604-2

**Published:** 2019-01-17

**Authors:** Joanna Stachecka, Joanna Nowacka-Woszuk, Pawel A. Kolodziejski, Izabela Szczerbal

**Affiliations:** 1 Department of Genetics and Animal Breeding, Poznan University of Life Sciences, Wolynska 33, 60-637 Poznan, Poland; 2 Department of Animal Physiology and Biochemistry, Poznan University of Life Sciences, Wolynska 35, 60-637 Poznan, Poland

**Keywords:** Adipocytes, Allele, Mesenchymal stem cells, Nuclear architecture, Pig, RNA-FISH

## Abstract

Proper expression of the *PPARG* gene, which encodes a key transcription factor of adipogenesis, is indispensable in the formation of mature adipocytes. The positioning of a gene within the nuclear space has been implicated in gene regulation. We here report on the significance of the *PPARG* gene’s nuclear positioning for its activity during in vitro adipogenesis in the pig. We used an established system of differentiation of mesenchymal stem cells derived from bone marrow and adipose tissue into adipocytes. The differentiation process was carried out for 7 days, and the cells were examined using the 3D DNA/immuno-FISH and RNA/DNA-FISH approaches. *PPARG* transcript level was measured using real-time PCR, and PPARγ activity was detected with colorimetric assay. Changes in the nuclear location of the *PPARG* gene were observed when we compared undifferentiated mesenchymal stem cells with mature adipocytes. The gene moved from the nuclear periphery to the nuclear center as its transcriptional activity increased. The RNA/DNA-FISH approach shows that differences in primary transcript production correlated with the allele’s nuclear positioning. Transcriptionally active alleles preferentially occupy the central part of the nucleus, while inactive alleles are found on the nuclear periphery. We also show that transcription of *PPARG* begins with one allele, but that both alleles are active in later stages of differentiation. Our results provide evidence that functionally distinct alleles of the *PPARG* gene are positioned in different parts of the cell nucleus. This confirms the importance of nuclear architecture to the regulation of *PPARG* gene transcription, and thus to the fate of the adipose cell.

**Electronic supplementary material:**

The online version of this article (10.1007/s10577-019-09604-2) contains supplementary material, which is available to authorized users.

## Introduction

Adipogenesis, the formation of fat cells, is regulated by a complex and orchestrated gene expression program (Lowe et al. 2011). A number of positive and negative transcription factors of adipocyte differentiation have been identified (Farmer 2006), and a central role is played among them by peroxisome proliferator-activated receptor γ (PPARγ), a ligand-activated transcription factor that belongs to the nuclear receptor superfamily. This factor has been recognized as the master regulator of adipogenesis, since no other factor has been discovered to promote this process in the absence of PPARγ (Rosen and MacDougald 2006). Apart from adipocyte differentiation, PPARγ also regulates adipocyte metabolism in mature adipocyte cells and insulin sensitivity (Lehrke and Lazar 2005; Siersbæk et al. 2010). PPARγ is encoded by the *PPARG* gene, which is highly expressed in adipose tissues, and whose expression increases during adipogenesis (Lee and Ge 2014). PPARγ upregulates genes involved in fatty acid metabolism and triglyceride storage and is consequently involved in maintaining the adipocyte phenotype (Ruschke et al. 2010).

Given the importance of PPARγ in the development of adipose tissue, understanding the molecular mechanisms that regulate its expression is highly important in the context of human obesity and obesity-related disorders (Shao et al. 2016). Adiposity is also of great interest in domestic animals, since proper functioning of the adipose tissue determines animal health, neonatal survival, reproductive ability, postnatal growth, and production efficiency (Louveau et al. 2016; Stachowiak et al. 2016). The transcriptional regulation of *PPARG* during adipogenesis is well understood, and many positive and negative factors, as well as signaling pathways, have been described (Rosen and MacDougald 2006; Farmer 2006; Mota de Sá et al. 2017). *PPARG* expression is also controlled by epigenetic mechanisms, including DNA methylation, histone modifications, and chromatin remodeling (Fujiki et al. 2009; Sugii and Evans 2011; Lee and Ge 2014). Apart from these mechanisms, it has been postulated that nuclear organization may play a role in gene regulation (Fraser and Bickmore 2007; Fedorova and Zink 2008). Mammalian genomes are hierarchically organized in the interphase nucleus, starting from the chromosome territories (Cremer and Cremer 2010) and the A (active) and B (inactive) compartments (Lieberman-Aiden et al. 2009), through topologically associating domains (TADs) and smaller sub-TADs (Dixon et al. 2012; Phillips-Cremins et al. 2013), which are stabilized by cohesin and CCCTC-binding factor (CTCF) (Rao et al. 2014; Sanborn et al. 2015). It has been postulated that this higher-order organization of the genome has some functional relevance.

It has been well established that chromosome territories, chromatin domains, and genes occupy preferential locations within the three-dimensional (3D) nuclear space and that their arrangement may affect gene expression (Cremer and Cremer 2010; Ferrai et al. 2010). The positioning of genes toward the nuclear periphery or the nuclear interior, the location of one locus relative to another, and their associations with different nuclear components (such as the chromosome territory, the nuclear envelope, and the nucleolus) and bodies (including nuclear speckles and transcription factories) are related to transcriptional status and can lead to their activation or repression (Meaburn 2016). In three-dimensional nuclear space, the positions of the chromosome territories are correlated with gene density: gene-rich chromosomes are located more centrally, while gene-poor chromosomes are usually located closer to the nuclear periphery (Bolzer et al. 2005). The differential positioning of active and silent genes within the nuclear space has also been observed, with active genes tending to be located in the nuclear interior and inactive genes on the nuclear periphery (Meister et al. 2010). Changes in nuclear organization and nuclear architecture are characteristic of cellular differentiation and development processes, including adipogenesis (Charó et al. 2016; Stachecka et al. 2018). Previous studies on a porcine in vitro model of adipocyte differentiation have shown that key adipogenic genes are repositioned from the nuclear periphery to the nuclear interior without relocation of the chromosome territories (Szczerbal et al. 2009). These gene relocations correspond with transcriptional activity. This also concerns the *PPARG* gene, located on porcine chromosome 13 (SSC13), which moves from the nuclear periphery to the nuclear interior during the differentiation of mesenchymal stem cells (MSC) into adipocytes.

The repositioning of *PPARG* during adipogenesis has not been investigated in greater detail on the cellular level. The study described here thus aimed to comprehensively examine the effects of the nuclear positioning of *PPARG* on its expression. The study was performed on the domestic pig (*Sus scrofa*), which is not only an important livestock species, but is also used as a model organism for human diseases, including obesity. We compared two in vitro adipogenic differentiation systems derived from porcine mesenchymal stem cells isolated from bone marrow (BM-MSC) and adipose tissue (AD-MSC). Using both cellular methods (3D DNA/immuno-FISH and DNA/RNA-FISH) and molecular methods (real-time PCR and colorimetric assay), we evaluated how the nuclear positioning of *PPARG* gene alleles affects their expression over subsequent days of adipogenesis.

## Materials and methods

### Cell culture

Porcine mesenchymal stem cells were isolated from adipose tissue and bone marrow from a 3-month-old Polish Large White pig, as described by Kociucka et al. (2016). The cells were cultured in advanced DMEM medium (Gibco) supplemented with 10% FBS (Sigma-Aldrich), 5 ng/ml FGF-2 (PromoKine), 2 mM L-Glutamine (PAA), 1 mM 2-mercaptoethanol (Sigma-Aldrich), 1 × antibiotic antimycotic solution (Sigma Aldrich), and MEM NEAA (Thermo Fisher Scientific) at 37 °C with 5% CO_2_ supplementation. To induce adipogenic differentiation, the cells were grown to confluency and were cultured with adipogenic differentiation medium composed of advanced DMEM (Gibco) with 10% (*v*/*v*) FBS (Sigma), 1 × antibiotic antimycotic solution (Sigma Aldrich), MEM NEAA (Thermo Fisher), 5 ng FGF-2 (PromoKine), 1 × linoleic acid albumin (Sigma-Aldrich), 1 × ITS (Sigma-Aldrich), 1 μM dexamethasone (Sigma-Aldrich), 100 μM indomethacin (Sigma-Aldrich), and 50 mM IBMX (Sigma-Aldrich). The differentiation process was allowed to proceed for 7 days and lipid droplet formation was examined using a phase-contrast microscope (TS100 Eclipse, Nikon).

### Oil Red O staining

Lipid accumulation was monitored with the use of Oil Red O staining. Briefly, cells were fixed with 4% paraformaldehyde in PBS (*w*/*v*) for 15 min at room temperature (RT). The cells were then rinsed with double distilled water and incubated with 0.3% Oil Red O (Sigma-Aldrich) solution in 2-propanol diluted in water (3:2 *v*/*v*) at RT for 1 h. After extensive rinsing with double-distilled water, the cells were left in PBS and examined using a phase-contrast microscope (TS100 Eclipse, Nikon).

### Real-time PCR

Total RNA was isolated from cells on each day of differentiation using TriPure Isolation Reagent (Roche Diagnostic), following the manufacturer’s instructions. The concentration and purity of the RNA samples were determined using a NanoDrop spectrophotometer (Thermo Scientific). A Transcriptor High Fidelity cDNA Synthesis Kit (Roche Diagnostic) was used to reverse transcribe 2 μg RNA. The *PPARG* transcript level was conducted using a LightCycler 480 SYBR Green I Master kit (Roche Diagnostic) on a LightCycler 480 II (Roche Life Science). The analysis was performed in duplicate for each sample. Relative transcript quantification was carried out using the 2-ΔΔCT method (Livak and Schmittgen 2001) with undifferentiated cells (day 0) as a calibrator. The ribosomal protein L27 (*RPL27*) gene was used as a reference gene. The primer sequences are provided in Supplementary Table 1.

### Protein analysis

Nuclear protein extraction was performed using a Nuclear Extraction Kit (Abcam), following the manufacturer’s instructions. Nuclear extracts were collected on each day of adipogenesis. Protein concentration was measured with a Qubit Protein Assay Kit (Thermo Fisher Scientific) on a Qubit 2.0 Fluorometer (Invitrogen). PPARγ DNA-binding affinity was assessed using a PPARγ transcription factor assay kit (Abcam). The experiment was performed twice—initially in duplicate and then in triplicate. The protein concentrations were equalized and 10 μl of nuclear extract was added to the wells, coated with a specific DNA sequence containing the peroxisome proliferator response element, and incubated overnight at 4 °C. Washing, incubation with antibodies (primary polyclonal anti-PPARγ antibody and goat anti-rabbit HRP conjugate secondary antibody), and color development procedures were performed in line with the manufacturer’s protocol. The absorbance was read at 450 nm using a Synergy2 device (Biotek).

### Hybridization probe

A probe for detecting the *PPARG* gene was derived from the PigE BAC library (Anderson et al. 2000) (http://www.arkgenomics.org/clonesVectors/). The BAC clone (PigE-86P19) was selected to span only the sequence of the gene of interest. DNA from the BAC clone was isolated by alkaline lysis and labeled by the random-priming method with biotin-16-dUTP (Roche Diagnostic) or digoxigenin-11-dUTP (Roche Diagnostic) using a BioPrimer Array CGH genomic labeling system (Thermo Fisher Scientific). The specificity of the probe was verified by PCR using specific primers (Suppl. Table 1) and 2D fluorescence in situ hybridization (2D-FISH) on metaphase chromosomes (Suppl. Fig. 1). Slides were analyzed under a Nikon E600 Eclipse fluorescence microscope.

### 3D DNA/immuno-FISH

Detection of DNA target and nuclear protein was performed in line with the protocol described by Solovei and Cremer (2010), with slight modifications. Cells were grown directly on glass slides. Cells from each day of adipocyte differentiation were fixed in 4% paraformaldehyde in PBS (*w*/*v*) for 10 min. The 3D preserved cells were then incubated in 20% glycerol in PBS (*v*/*v*) and exposed to seven repeated freeze–thaw cycles in liquid nitrogen. The cells were treated with 0.1 M HCl for 5 min and equilibrated in 50% formamide in 2 × SSC. The probe was denatured at 70 °C for 10 min and preannealed at 37 °C for 10 min. The nuclei were denatured at 75 °C in 70% formamide in 2 × SSC for 3 min and in 50% formamide in 2 × SSC for 1 min. Hybridization was performed in a humid chamber at 37 °C for 2 days. After hybridization, the slides were washed three times in 50% formamide in 2 × SSC for 6 min and three times in 2 × SSC for 6 min at 42 °C. The slides were equilibrated in 4 × SSC/0.05% Tween-20, and then blocked with 3% BSA in 4 × SSC/0.05% Tween-20 for 30 min at RT. Detection was performed using antidigoxigenin–fluorescein Fab fragments (Roche Diagnostic) at a dilution of 1:100. The slides were washed three times in 4 × SSC/0.05% for 6 min at 42 °C and 3 times for 5 min in PBS at RT. In the next step, cells were subjected to immunostaining. Primary antibody against lamin A/C (monoclonal anti-lamin A/C produced in mouse, Sigma-Aldrich) was applied on slides at a dilution of 1:100 and incubated overnight at 4 °C. The slides were then washed three times in PBS and incubated with secondary antibody (anti-mouse IgG (whole molecule)-TRITC antibody produced in rabbit, Sigma-Aldrich) diluted 1:200 for 1 h at RT. This was followed by three washes in PBS for 5 min. The nuclei were counterstained with DAPI in Vectashield mounting medium (Vector Laboratories).

### 3D RNA/DNA-FISH

Sequential RNA and DNA detection by FISH was performed in line with the protocol described by Brown and Buckle (2010), with slight modifications. For this procedure, all buffers were prepared using DEPC-treated water. Cells growing directly on coverslips were fixed in 4% formaldehyde and 5% glacial acetic acid in 1 × saline for 20 min and washed three times in PBS (*w*/*v*). The coverslips were stored in 70% EtOH at − 20 °C. Before hybridization, the slides were equilibrated in Tris/saline solution and permeabilized with 0.02% pepsin solution for 4 min at 37 °C. The coverslips were then washed in DEPC-treated water, fixed in 3.7% formaldehyde solution for 4 min, and washed with PBS (*w*/*v*), before being dehydrated with 70% EtOH, 90% EtOH, 100% EtOH, and air-dried. The probe was denatured at 90 °C for 8 min and preannealed at 37 °C for 10 min. Hybridization was performed overnight in a humid chamber at 37 °C, after which the coverslips were washed three times with 2 × SSC, followed by two washes in Tris/saline/Tween-20 solution. Then, cells were blocked in 1.35% blocking solution (ELISA blocking reagent, Roche Diagnostic) for 30 min at RT. Detection was performed using Cy3-Streptavidin (GE HealthCare) diluted 1:200. The cells were then fixed in 3.7% formaldehyde solution for 4 min and washed with PBS (*w*/*v*). DNA hybridization was performed directly after RNA detection. The nuclei were denatured at 75 °C in 70% formamide in 2 × SSC for 3 min and in 50% formamide in 2 × SSC for 1 min. The probe was denatured at 70 °C for 10 min and preannealed at 37 °C for 10 min. Hybridization was performed in a humid chamber at 37 °C for 2 days. After hybridization, the coverslips were washed three times in 50% formamide in 2 × SSC for 6 min at 42 °C and three times in 2 × SSC for 6 min at 42 °C. The coverslips were equilibrated in 4 × SSC/0.1% Tween-20 and blocked with 3% BSA in 4 × SSC/0.05% Tween-20 for 30 min at RT. Detection of DNA targets was performed with antidigoxigenin-fluorescein Fab fragments (Roche Diagnostic) diluted 1:100. Washes were performed three times in 4 × SSC/0.05% Tween-20 for 6 min at 42 °C and three times for 5 min in PBS at RT. Nuclei were counterstained with DAPI in Vectashield mounting medium (Vector Laboratories).

### Confocal microscopy and image analysis

The cells were examined under an LSM 880 confocal microscope with Airyscan (Carl Zeiss). The slides were first examined under a 40×/1.2 Plan-Apochromat objective with visible light and laser excitation line 405 for DAPI in order to detect cells with lipid droplet accumulation (Suppl. Fig. 2) and to identify the cells undergoing adipocyte differentiation. Z-stacks were acquired with a 63×/1.4 NA Plan-Apochromat oil objective at a step size of 0.2 μm and consisted of approximately 35–50 slices. The pixel size was 71 nm by 71 nm by 200 nm. The images were acquired by lasers with three excitation lines: 543 nm for Cy3 and TRITC, 488 nm for FITC, and 405 nm for DAPI. The confocal microscope images were taken with an Airyscan detector in fast scan mode using Zeiss Zen Black software. The pinhole, filters, and objectives were kept at constant settings throughout the examination of all slides. The image stacks were processed by Airyscan processing to increase the signal-to-noise ratio and image resolution. Approximately 200–400 nuclei from AD-MSCs and BM-MSCs were analyzed on each of the 7 days of adipogenesis using the TANGO plugin to FIJI (Ollion et al. 2013). The nuclei were segmented from the Z-stacks using a simple segmenter function and the hybridization signals were segmented using a hysteresis segmenter function. The radial position of the hybridization signals was evaluated as described by Kociucka et al. (2012). Briefly, the ratio of the measurement distance was calculated, where *R* was the distance from the signal to the center of the nucleus divided by the distance from the signal to the nuclear border. A value of *R* > 4.45 was taken for the nuclear periphery, 4.45 ≥ *R* ≥ 1.37 was considered an intermediate position, and *R* < 1.37 was considered an interior position.

### Statistical analysis

Statistical analysis was performed using SAS 9.4 software. All variables were tested for normal distribution. The differences in lipid droplet accumulation were tested using the test for two binomial parameters. The expression levels of *PPARG* gene and PPARγ protein activity were compared using Student’s *t* test. The differences in gene locations on each day of adipogenesis were tested using multiple comparisons following a Pearson Chi-square test. *P* values < 0.05 were considered significant.

## Results

**Fig. 1:**
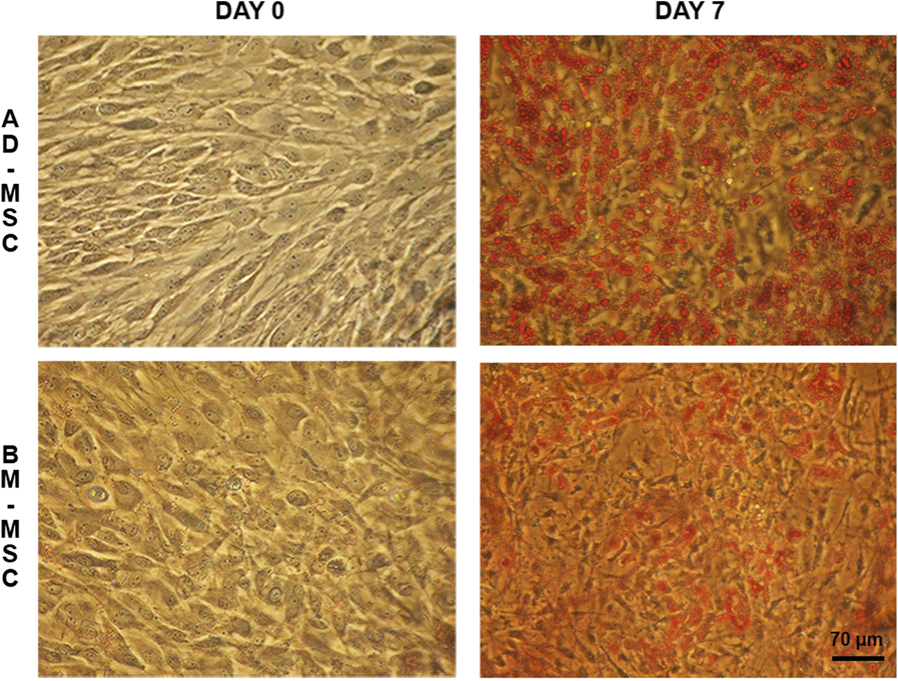
Adipogenic differentiation of porcine AD-MSCs (top panel) and BM-MSCs (bottom panel). Oil Red O staining was used to visualize lipid droplet formation on days 0 and 7 of adipogenesis. Note the increased accumulation of lipid droplets in AD-MSCs, as compared to BM-MSCs, on day 7

A system of in vitro differentiation of porcine MSC (pMSC) into adipocytes was used to characterize changes in the nuclear positioning of the *PPARG* locus in relation to its expression. pMSCs were derived from two different sources—adipose tissue (AD-MSC) and bone marrow (BM-MSC). As previously reported by Stachecka et al. (2018), these two cell types have different capacities for differentiation into adipocytes: AD-MSCs exhibit greater differentiation potential than BM-MSCs, which was evaluated by the accumulation of the amount of lipid droplets (Fig. 1). On day 7 of adipogenic differentiation, 62% of the cells exhibited visible lipid droplets after Oil Red O staining in AD-MSC, whereas this occurred only in 52% of BM-MSC cells (*P* < 0.05, *n* = 450). Adipogenesis was carried out for 7 days; on each day, beginning with undifferentiated cells on day 0, the activity and 3D location of the *PPARG* gene were determined.

**Fig. 2:**
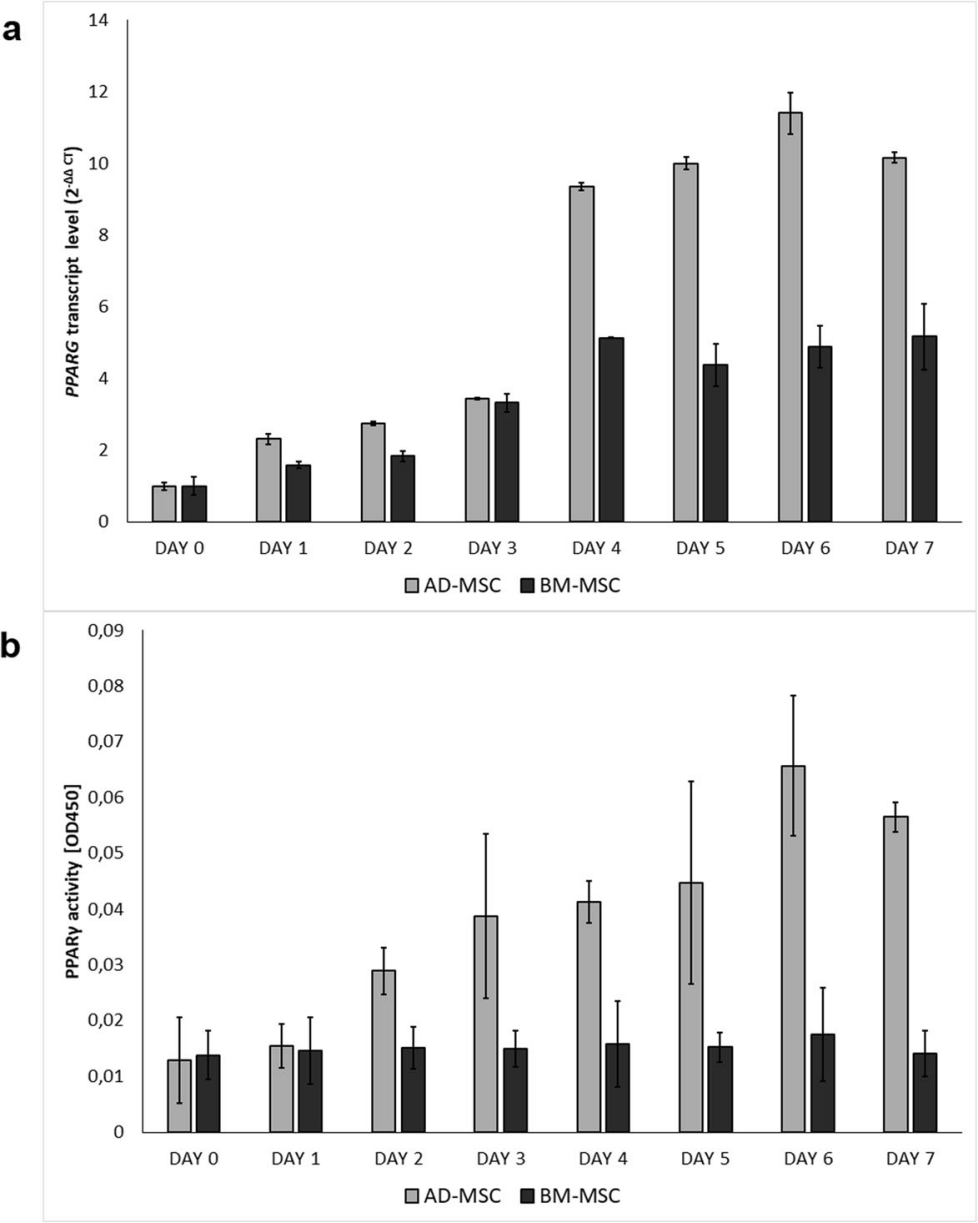
Expression of *PPARG* during porcine in vitro adipogenesis. **a** Relative mRNA levels on subsequent days of differentiation in AD-MSC and BM-MSC systems, calculated for the *RPL27* gene and using day 0 as a calibrator (day 0 *n*_AD-MSC_ = 16, *n*_BM-MSC_ = 16). Error bars show SDs. **b** PPARγ activity on subsequent days of porcine in vitro adipogenesis (*n*_AD-MSC_ = 40, *n*_BM-MSC_ = 40). Error bars show SDs

The *PPARG* relative transcript level was measured using the AD-MSC and BM-MSC systems. We observed that *PPARG* was weakly transcribed in both type of undifferentiated cells, and its transcription increased through subsequent days of adipogenesis (Fig. 2a). *PPARG* transcript was less abundant in BM-MSCs than in AD-MSCs (*P* < 0.05). PPARγ activity was measured as protein binding affinity in the colorimetric assay. It was observed that its activity in the AD-MSC system increased in subsequent days of differentiation, with the highest activity found on day 6 (Fig. 2b, *P* < 0.05). Surprisingly, no statistically significant differences were found in PPARγ activity during differentiation in BM-MSC system. Similarly to the transcript level, the protein activity was also lower in the BM-MSCs than in the AD-MSCs (*P* < 0.05). These results indicate that the expression of *PPARG* positively correlates with the adipogenic capacity observed on the cellular level through the formation of mature adipocytes in the AD-MSC and BM-MSC.

**Fig. 3:**
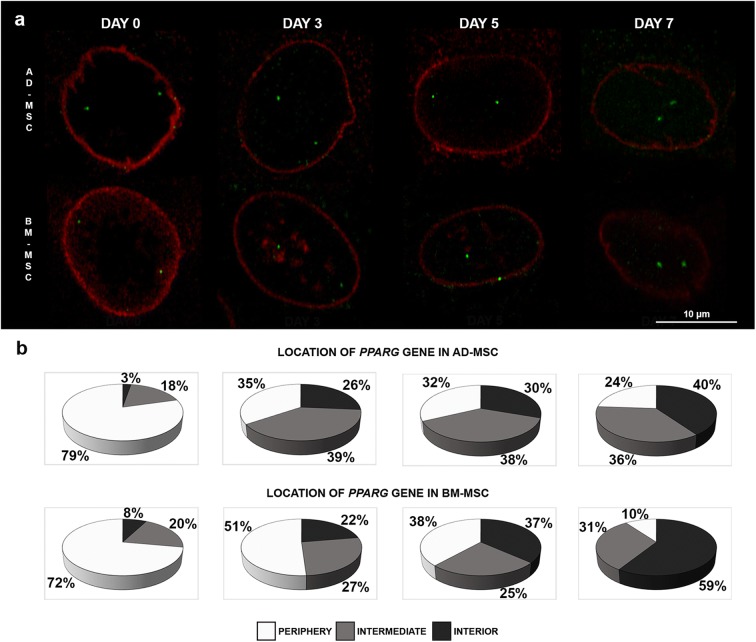
Nuclear positioning of the *PPARG* gene during in vitro porcine adipogenesis. **a** 3D DNA/immuno-FISH was used to visualize the *PPARG* locus (green) in relation to the nuclear lamina (red). The images are representative confocal slices. Reduced lamin A/C expression is characteristic of adipocyte differentiation. **b** Analysis of the distribution of the *PPARG* gene in the nuclear space of AD-MSCs and BM-MSCs on days 0, 3, 5, and 7 of adipogenesis. The percentage of hybridization signals located in the nuclear interior, intermediate, and periphery is shown (*n*_AD-MSC_ = 469, *n*_BM-MSC_ = 405, *P* < 0.05). Note the repositioning of the gene from nuclear periphery to nuclear center during differentiation

In the next step, we examined the radial positioning of the *PPARG* locus relative to the nuclear lamina using 3D DNA/immuno-FISH (Fig. 3a, Suppl. Fig. 3). We observed that, in undifferentiated cells, the gene preferentially occupied the nuclear edge: 79% of the gene signal in the AD-MSC system and 72% of the signal in the BM-MSC system had peripheral location (Fig. 3b). The gene was often localized near the nuclear lamina. In subsequent days of adipogenic differentiation, the location of *PPARG* changed to an intermediate position. On day 3 of differentiation, approximately one third of the *PPARG* gene signals showed an intermediate location (39% of gene signals in AD-MSC and 27% of gene signals in BM-MSC). In the subsequent days of adipogenesis, the percentage of cells with *PPARG* hybridization signals in the nuclear interior increased. On day 5, we observed that 30% of gene signals in AD-MSC and 37% in BM-MSC were in the central part of the cell nucleus; on day 7, this was the case for about 40% of gene signals in AD-MSC and 59% in BM-MSC (Fig. 3b). The changes we observed in *PPARG* nuclear positioning during the course of adipogenic differentiation in both systems were statistically significant (*n*_AD-MSC_ = 469, *n*_BM-MSC_ = 405 *P* < 0.05) (Suppl. Table 2).

**Fig. 4:**
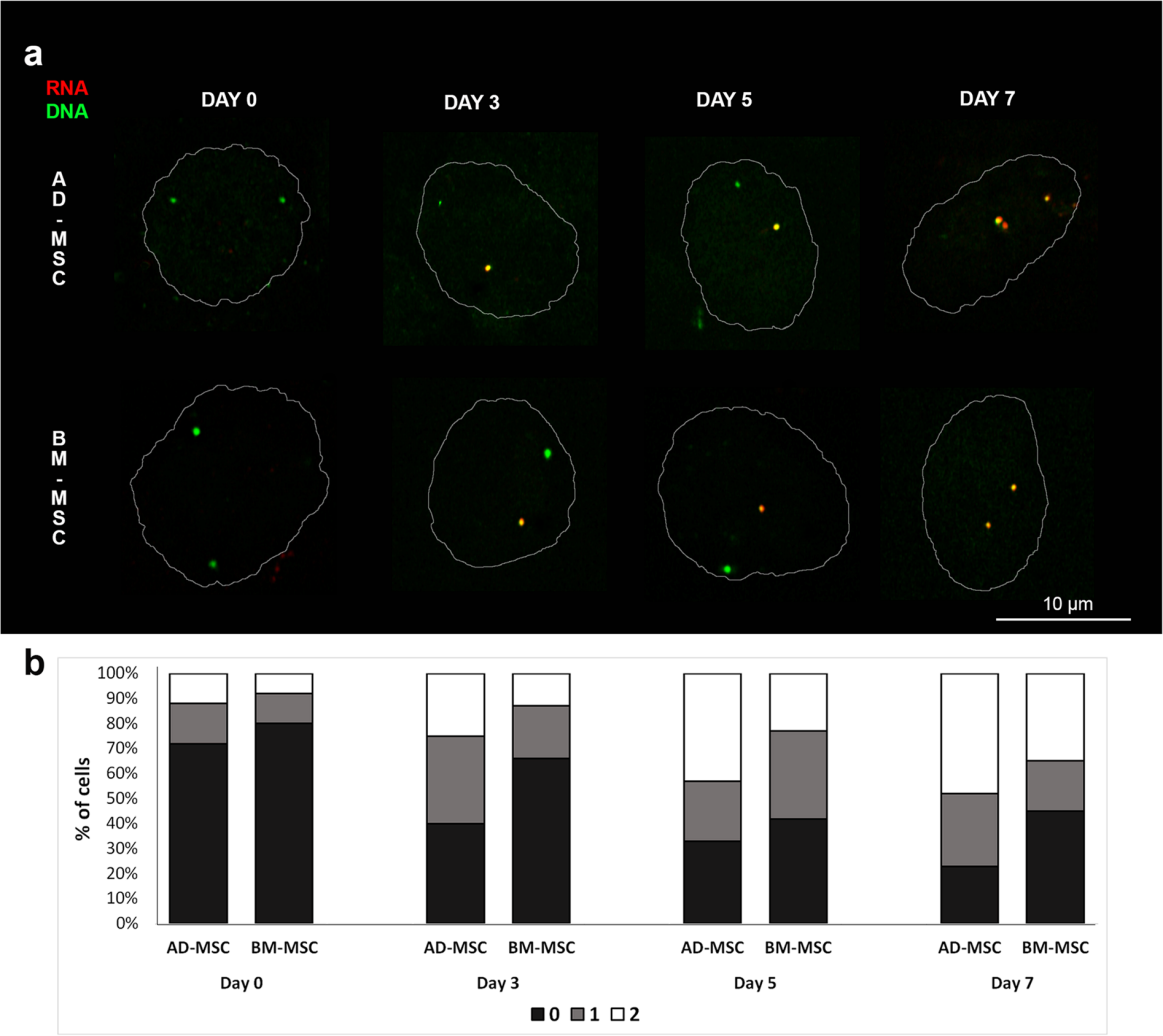
Detection of primary transcript of the *PPARG* gene during porcine in vitro adipogenesis. **a** 3D RNA/DNA-FISH was used to visualize pre-mRNA (red) and DNA locus (green). The limit of nuclear DAPI staining on confocal slices is shown by the white line. **b** The percentages of cells with 0, 1, or 2 active alleles on subsequent days of differentiation (*n*_AD-MSC_ = 400, *n*_BM-MSC_ = 400, *P* < 0.05). Note the preferential location of active alleles in the central part of nucleus and the unequal production of mRNA from both alleles

We used RNA/DNA-FISH to determine whether changes in the nuclear localization of *PPARG* are related to its transcriptional status. This technique allowed us to detect primary transcript production from a single allele (Fig. 4a, Suppl. Fig. 4). Alleles positive for the RNA hybridization signal were considered transcriptionally active, while alleles negative for the RNA hybridization signal were considered inactive. On day 0 of differentiation, 72% of the cells in AD-MSC and 80% in BM-MSC showed no pre-mRNA production from either allele. On subsequent days of adipogenesis, transcription began from a single allele: on day 3, 35% of the cells in AD-MCS and 21% in BM-MSC had one active allele. We observed that transcription from both alleles began earlier in AD-MSC than in BM-MSC (43% of cells versus 23%). As differentiation proceeded, both alleles became active: on day 7, 48% of cells in AD-MSC and 35% in BM-MSC possessed two active alleles (Fig. 4b, *n*_AD-MSC_ = 400, *n*_BM-MSC_ = 400).

**Fig. 5:**
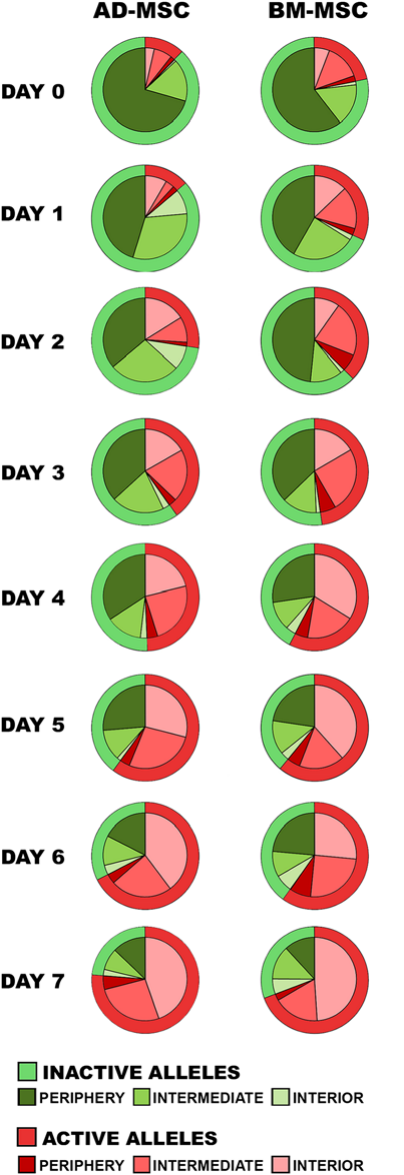
Distribution of transcriptionally active and inactive alleles of the *PPARG* gene in the nuclear interior, intermediate, and periphery in the AD-MSC and BM-MSC systems over subsequent days of adipogenic differentiation (*n*_AD-MSC_ = 1955, *n*_BM-MSC_ = 1461, *P* < 0.05)

We next examined how the radial position of the *PPARG* alleles in nuclear space affects their transcriptional activity. We found that active and inactive alleles had different nuclear positions in the AD-MSC and BM-MSC systems (Fig. 5, Suppl. Table 3). A peripheral location was characteristic of inactive alleles. In undifferentiated cells, 80.6% of inactive alleles in AD-MSC and 77.4% in BM-MSC were located on the nuclear periphery. On the other hand, only 7.0% of active alleles in AD-MSC and 3.6% in BM-MSC had peripheral locations on day 7. Throughout adipogenic differentiation, as more alleles became active, we observed that they had central and intermediate nuclear positions. By day 7, 58.6% of the active alleles in AD-MSC had central localizations and 34.4% had intermediate localizations; in BM-MSC, 70.9% of the active alleles had a central position and 25.5% were located in intermediate positions. Significant differences in the nuclear location of active and inactive alleles were visible on each day of adipogenic differentiation in both of the examined systems, with active alleles being localized in the central and intermediate nuclear positions, and inactive ones on the nuclear periphery (*n*_AD-MSC_ = 1955, *n*_BM-MSC_ = 1461, *P* < 0.05, Fig. 5, Suppl. Table 3). We also compared the nuclear position of active and inactive alleles in all the examined cells with only one transcriptionally active allele. We observed that active alleles preferentially occupied central and intermediate positions. In AD-MSC, 44.8% of active alleles had central positions and 48.5% had intermediate positions, while in BM-MSC, 44.3% of active alleles had central positions and 46.9% had intermediate positions. Inactive alleles were located preferentially on the nuclear periphery (58.7% in AD-MSC and 61.1% in BM-MSC) (Supp. Fig. 5, Suppl. Table 4). Our results indicate that there is a relationship between *PPARG* gene activity and its nuclear positioning.

## Discussion

In this study, we have described the detailed behavior of the locus of the *PPARG* gene in the nuclear space on each of the 7 days of differentiation of porcine mesenchymal stem cells into adipocytes. The gene was upregulated during differentiation. Its transcript level was higher in AD-MSC than in BM-MSC, which can be explained by the different adipogenic potential of these two types of MSCs. We have shown that higher expression of the *PPARG* gene enhances adipogenesis in AD-MSC. Similar results to those in this study have also been reported in humans (Liu et al. 2017); the authors of that report examined the adipogenic ability of MSCs isolated from umbilical cords, adipose tissue, and bone marrow and found AD-MSCs to display the highest potential to undergo adipogenesis—which could be an effect of the different expression pattern of the miR-301b~miR-130b–PPARγ axis. Also, overexpression of the *PPARG* gene enhanced and accelerated the adipogenic differentiation of hemangioma-derived mesenchymal stem cells (Hem-MSCs) (Yuan et al. 2017). Correlation between the *PPARG* gene’s transcriptional and protein activity was observed only in the AD-MSC system. That lack of difference in the activity of *PPARG* on subsequent days of adipogenesis in BM-MC can be explained by the small amount of PPARγ in the nuclear extract of these cell types, and the limitations involved in detecting it using immunoassays, as has been previously reported by Chan and Cipolla (2012).

The repositioning of the *PPARG* gene from the nuclear periphery to the nuclear center corresponded with its transcriptional activity. This is in agreement with observations in a previous study, where only three time points (days 0, 7, and 14 of adipogenesis) were evaluated by RT-PCR (Szczerbal et al. 2009). Here, using more advanced techniques, we compared two different systems of adipogenic differentiation (AD-MSC and BM-MSC) and detected gradual changes in *PPARG* repositioning and transcript level on each day of adipogenesis. Along with the progression of adipogenesis, the number of cells with a centrally located gene locus significantly increased. This indicates that changes in *PPARG* nuclear relocation are a universal phenomenon, independent of the experiment and of the types of cell used as progenitors.

The nuclear periphery is generally considered to be a transcriptionally less active part of the cell nucleus. Genomic regions in contact with the nuclear lamina, termed lamina-associated domains (LADs), have been linked to gene repression (van Steensel and Belmont 2017). Two classes of LAD have been distinguished: constitutive LADs that interact with the lamina in all cell types, and facultative LADs, which are cell-type specific (Meuleman et al. 2013). A number of repositioning events from the nuclear periphery to the nuclear interior have been described, mainly during the processes of differentiation and development, including erythropoiesis, lymphopoiesis, and neuronal differentiation (Kosak et al. 2002; Kim et al. 2004; Ragoczy et al. 2006; Williams et al. 2006; Peric-Hupkes et al. 2010). Other cellular processes, such as senescence, have been shown to be characterized by massive changes in LAD organization (Lenain et al. 2017). Our previous study also showed that adipogenesis is characterized by a reorganization of nuclear lamina (Stachecka et al. 2018). The experimental targeting of genes to the nuclear periphery has also been shown to result in transcriptional repression (Finlan et al. 2008; Reddy et al. 2008); however, not all genes were subjected to these mechanisms (Kumaran and Spector 2008). It has been also demonstrated that the relocation of genes from the nuclear envelope to the nuclear interior during differentiation is driven by chromatin remodeling, rather than by transcription per se (Therizols et al. 2014). Analysis of the genome-wide chromatin state in two models of adipogenesis (murine and human) has shown that one of the promoters (P2) of the *PPARG* gene dissociates from the lamina and acquires H3K4me3 modification as it is upregulated in adipocytes (Mikkelsen et al. 2010; Lund and Collas 2013). Unfortunately, such analysis was not performed so far for the porcine *PPARG* gene. A study of the nuclear arrangement of other genes important in lipid metabolism in porcine adipocytes in fat tissue showed that the correlation between transcription level and 3D location existed only for some genes (Kociucka et al. 2012). In addition, the transcriptional activation of genes by hormone addition or bacterial infection did not induce changes in the positioning of genes in the nuclear space (Kocanova et al. 2010; Hakim et al. 2011; Solinhac et al. 2011). There are also examples of genes in cancer cells that have been found to alter their nuclear position without changes in expression (Meaburn and Misteli 2008). This indicates that the relationship between the radial positioning of genes and their transcriptional status is complex. Our study has confirmed previous suggestions that nuclear repositioning is mainly typical of genes that switch from the silent state to the active state during differentiation processes (Joffe et al. 2010).

We have here demonstrated the allele-specific radial nuclear positioning of *PPARG* during in vitro adipogenesis. In mature adipocytes, the active alleles preferentially occupied the interior of the nucleus. On day 7 of differentiation, only 3.6–7.0% of active alleles were located on the nuclear periphery. Different positioning of the alleles in nuclear space was also reported by Takizawa et al. (2008), who studied the monoallelically expressed *GFAP* gene and showed that the active allele was more internally localized than the nonexpressed allele. Genes that undergo random autosomal monoallelic expression are good models for studying the link between nuclear architecture and gene expression. It has been shown that nuclear positioning is important for the monoallelic expression of immunoglobulin (Skok et al. 2001) and olfactory receptor (Clowney et al. 2012) genes, but this has not been demonstrated for monoallelic expressed genes in mouse embryonic stem cells or neural progenitor cells (Eckersley-Maslin et al. 2014), as the researcher did not find a preferential location of inactive alleles at the nuclear periphery or near heterochromatic foci.

The *PPARG* gene undergoes biallelic expression. Analysis of the allele-specific expression of this gene in porcine fat tissues has shown that both alleles are expressed at similar levels (Stachowiak et al. 2018). However, the use of RNA/DNA-FISH allowed us to detect temporal differences in *PPARG* transcript production. In spite of the biallelic nature of *PPARG* gene expression, the transcript is initially produced from only one allele. Such subtle differences in transcript production and accumulation could easily be detected with microscopic techniques. It is well established that FISH-based methods have many advantages in studies of nuclear architecture—for example, RNA-FISH allows the detection of the spatial and temporal organization of gene transcription in individual cells and the identification of intercellular variation. However, a limitation of such techniques is their low throughput; thus, they are usually used only to analyze candidate genes (Meaburn 2016; Brown and Buckle 2010).

The spatial positioning of genomic loci can be also studied with biochemical methods originating from the chromatin conformation capture technique (3C), such as 3C, 4C, 5C, and Hi-C (Dekker and Misteli 2015). These approaches have also been used to study the *PPARG* gene. It has been shown that, during the differentiation of mouse 3T3-L1 and C3H10T1/2 cell lines into adipocytes, the *PPARG* locus makes long-range chromatin interactions with selected tissue-specific loci, encoding adipokines and lipid-droplet-associated proteins, such as perlipin, adiponectin, and leptin (LeBlanc et al. 2014), or creating an adipogenic-specific nuclear subcompartment referred as a “hub” with the *Lpin1* gene (Sarusi Portuguez et al. 2017). To date, chromatin interaction studies on a genome-wide level have not been performed for porcine adipogenesis. However, it can be anticipated that the observed repositioning of the *PPARG* gene is associated with its interaction with other chromosomal regions or nuclear subcompartments, as has been previously observed in the case of SC-35 domains (Szczerbal and Bridger 2010).

There is no doubt that the position of genes in nuclear space is not random. However, it is still not clear whether gene repositioning events are necessary for activation or are merely a consequence of the gene expression program (Shachar and Misteli 2017). The molecular mechanisms governing the spatial reposition of genes have not yet been fully elucidated. However, based on the results obtained so far, it seems that these mechanisms may differ for different loci and may depend on nuclear envelope proteins (Solovei et al. 2013), DNA sequence (Zullo et al. 2012), histone modifications (Towbin et al. 2012), and replication (Shachar et al. 2015). It can be anticipated that the use of new technologies, such as single-cell Hi-C and CRISPR-Cas9 methods (Ramani et al. 2017; Morgan et al. 2017), will lead to deeper insights into the mechanisms that determine the spatial positions of genes and their functional consequence. Understanding how nuclear architecture contributes to the expression of adipogenic genes and the establishment of mature adipocytes is important for both biomedical and animal sciences.

## Electronic supplementary material

ESM 1 (DOCX 4553 kb)

## Abbreviations

3D: three-dimensional
AD-MSC: adipose-tissue-derived mesenchymal stem cells
BM-MSC: bone-marrow-derived mesenchymal stem cells
cDNA: complementary DNA
DAPI: 4′,6-diamidino-2-phenylindole
FISH: fluorescent in situ hybridization.
Hem-MSC: hemangioma-derived mesenchymal stem cells
PBS: phosphate buffered saline
PFA: paraformaldehyde
PPARG: peroxisome proliferator-activated receptor gamma
RT: room temperature
RT-PCR: reverse transcription polymerase chain reaction
SSC: saline sodium citrate buffer
SSC13: *Sus scrofa* chromosome 13
