# Supplementary material for: The importance of the nuclear positioning of the PPARG gene for its expression during porcine in vitro adipogenesis
Source: Chromosome Res. 2019 Jan 17;27(3):271–84. doi: 10.1007/s10577-019-09604-2 (PMC6733831; doi:10.1007/s10577-019-09604-2)
Supplement: Supplementary file 1 — (DOCX 4553 kb) [file 10577_2019_9604_MOESM1_ESM.docx]

**Supplementary Material**

**The importance of the nuclear positioning of the *PPARG* gene for its expression during porcine *in vitro* adipogenesis**

Joanna M. Stachecka,^1^ Joanna Nowacka-Woszuk,^1^ Pawel A. Kolodziejski,^2^ Izabela Szczerbal^1,*^

**
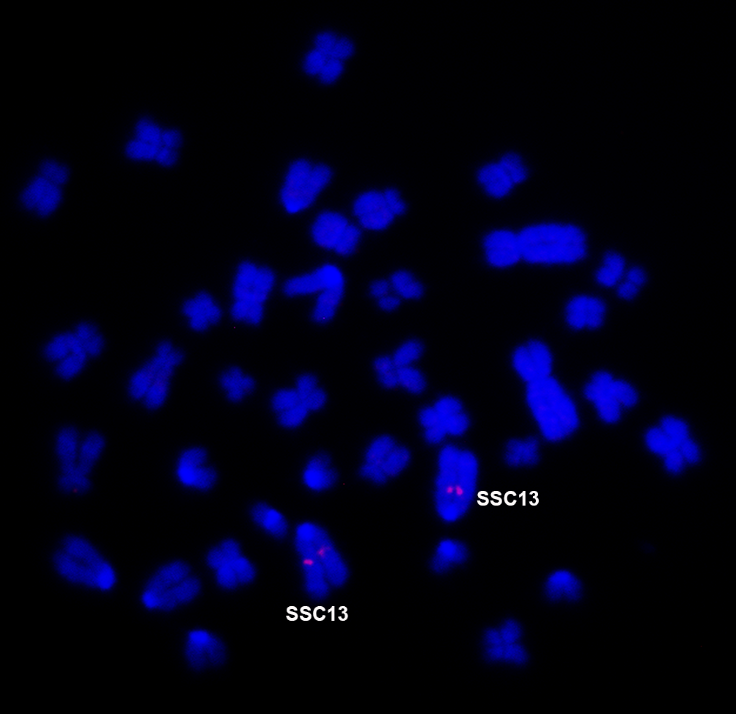
**

**Suppl. Fig. 1.** Representative image of the location of the *PPARG* probe on pig chromosome 13 (SSC13q24).


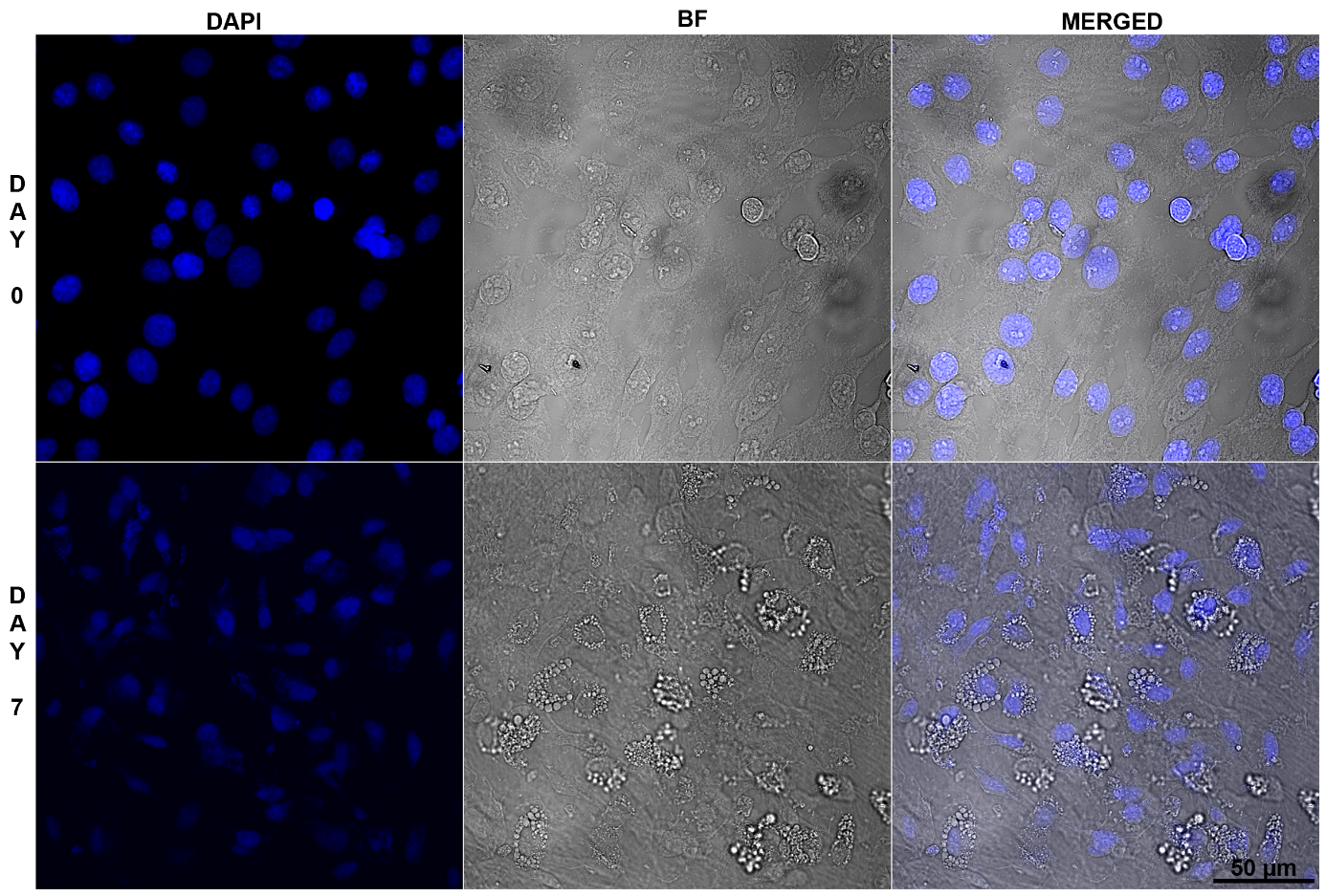


**Suppl. Fig. 2.** Identification of cells undergoing adipogenesis on slides for *in situ* hybridization. Representative images from days 0 (top panel) and 7 of differentiation (bottom panel) are presented. DAPI: chromatin staining; BF: bright-field. Note the accumulation of lipid droplets observed under BF channel on day 7 of adipogenesis.


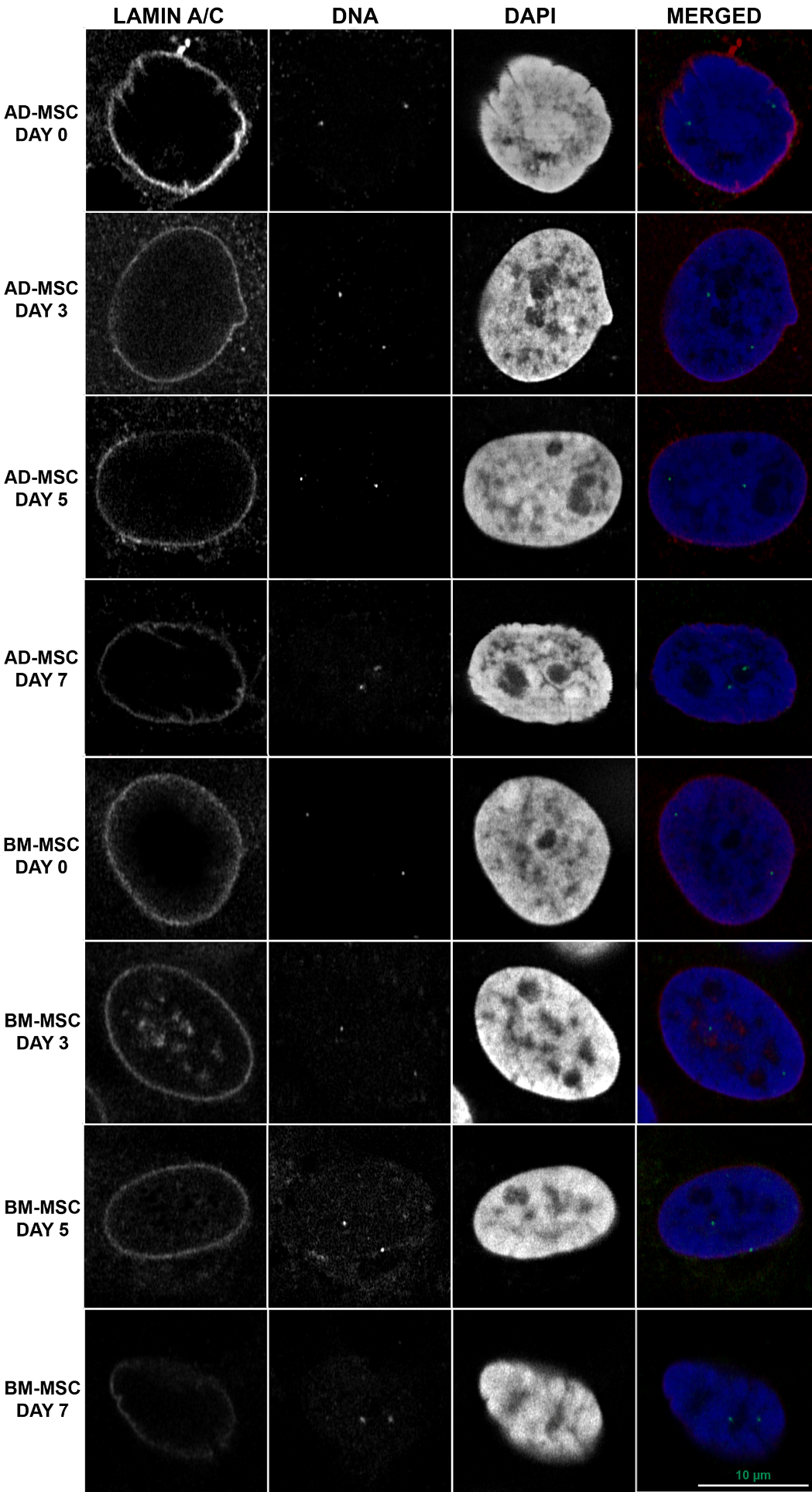


**Suppl. Fig. 3.** Unprocessed images from Figure 3. Presented in grayscale for lamin A/C (red – TRITC – 543 nm), *PPARG* locus (green – FITC – 488 nm), and chromatin (blue – DAPI – 405 nm).


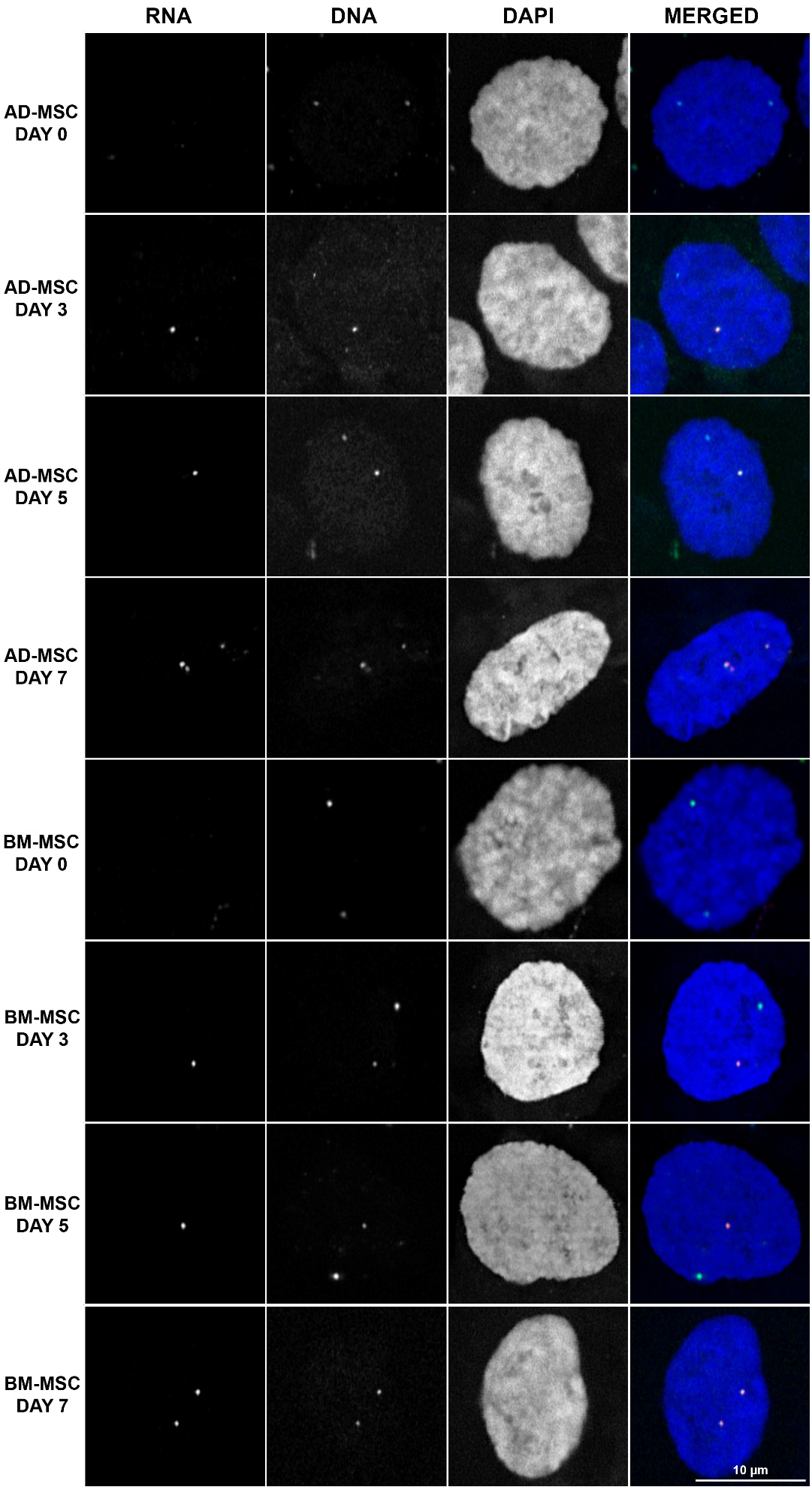


**Suppl. Fig. 4.** Unprocessed images from Figure 4. Presented in grayscale for RNA (red – Cy3 – 543 nm), DNA (green – FITC – 488 nm), and chromatin (blue - DAPI – 405 nm).


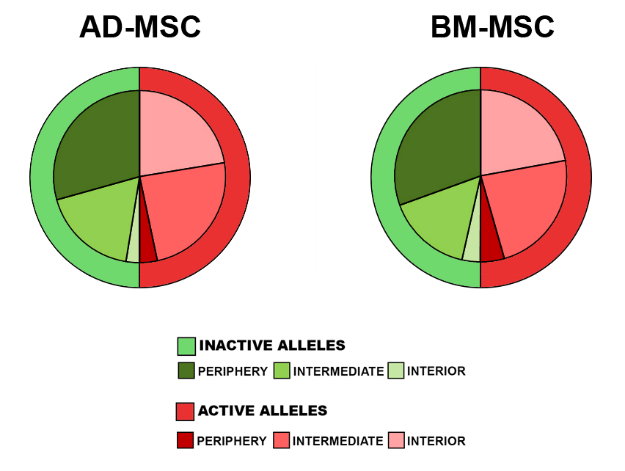


**Suppl. Fig. 5**. Nuclear distribution of transcriptionally active and inactive alleles within the nuclei, with only one active allele (n_AD-MSC_ = 649, n_BM-MSC_ = 542, P < 0.05).

**Suppl. Table 1.** PCR primers used in the study.

| Gene | Primer sequences | Amplicon length (bp) | Annealing  temperature (°C) | GenBank accession no. |
| --- | --- | --- | --- | --- |
| *PPARG*  (qPCR) | F: 5’ GCCCTTCACCACTGTTGATT 3’ | 210 | 60 | NM_214379.1 |
|  | R: 5’ GAGTTGGAAGGCTCTTCGTG 3’ |  |  |  |
| *RPL27*  (qPCR) | F: 5’ GCAAAGCGGTCATCGTAAA 3’ | 190 | 60 | NM_001097479.1 |
|  | R: 5’ CTTGTGGGCATGAGGTGAT 3’ |  |  |  |
| *PPARG*  (PCR) | F: 5’ GGTTTCTTCCGGAGGACTATCAG 3’ | 130 | 55 | NM_214379 |
|  | R: 5’ACATCCCCACAGCAAGGCATTTCT 3’ |  |  |  |

**Suppl. Table 2.** 3D location of the *PPARG* gene signals in the nuclear interior, intermediate and periphery during porcine *in vitro* adipogenesis. Number of analyzed cells for AD-MSC n_AD-MSC_=469, and for BM-MSC n_BM-MSC_=405.

|  | | | | | | | | | |
| --- | --- | --- | --- | --- | --- | --- | --- | --- | --- |
|  | **Location of gene signals Number of analyzed gene signals** | | | | | | | | |
|  |  | **Day 0**  **A*** | **Day 1**  **B** | **Day 2**  **B** | **Day 3**  **B** | **Day 4**  **B** | **Day 5**  **B,C** | **Day 6**  **C** | **Day 7**  **C** |
| AD-MSC | **INTERIOR** | 3 | 31 | 35 | 29 | 39 | 34 | 55 | 37 |
|  | **INTERMEDIATE** | 20 | 40 | 55 | 43 | 45 | 42 | 29 | 33 |
|  | **PERIPHERY** | 89 | 43 | 62 | 38 | 54 | 36 | 24 | 22 |
|  |  | **Day 0**  **A** | **Day 1**  **B** | **Day 2**  **A,B** | **Day 3**  **B,C** | **Day 4**  **C** | **Day 5**  **C** | **Day 6**  **C** | **Day 7**  **D** |
| BM-MSC | **INTERIOR** | 8 | 11 | 14 | 21 | 29 | 41 | 33 | 53 |
|  | **INTERMEDIATE** | 21 | 36 | 33 | 25 | 25 | 28 | 34 | 28 |
|  | **PERIPHERY** | 75 | 53 | 63 | 48 | 42 | 43 | 37 | 9 |

* Different capital letters (A, B, C, D) indicate statistically significant differences (P < 0.01), while the same capital letters indicate no statistically significant differences.

**Suppl. Table 3.** Nuclear location of active and inactive *PPARG* alleles during porcine *in vitro* adipogenesis. In total, 1955 cells were analyzed in the AD-MSC system and 1461 in BM-MSC.

|  | | | | | | | | | | |
| --- | --- | --- | --- | --- | --- | --- | --- | --- | --- | --- |
|  |  | **Location of signals % of observed *PPARG* alleles**  **(number of alleles is shown in brackets)** | | | | | | | | |
|  |  |  | **Day 0** | **Day 1** | **Day 2** | **Day 3** | **Day 4** | **Day 5** | **Day 6** | **Day 7** |
| AD-MSC | **TRANSCRIPTIONALLY ACTIVE** | **INTERIOR** | 28.6% (16) | 60.4% (32) | 58.5% (96) | 41.5% (108) | 42.9% (140) | 48.4% (108) | 59.1% (191) | 58.6% (143) |
|  |  | **INTERMEDIATE** | 57.1% (32) | 24.5% (13) | 36.0% (59) | 50.8% (132) | 48.8% (159) | 45.3% (101) | 35.3% (114) | 34.4% (84) |
|  |  | **PERIPHERY** | 14.3% (8) | 15.1% (8) | 15.5% (9) | 7.7% (20) | 8.3% (27) | 6.3% (14) | 5.6% (18) | 7.0% (17) |
|  | **TRANSCRIPTIONALLY INACTIVE** | **INTERIOR** | 0.8% (3) | 11.2% (37) | 12.7% (55) | 14.4% (17) | 5.1% (17) | 4.0% (6) | 11.5% (18) | 10.5% (8) |
|  |  | **INTERMEDIATE** | 18.7% (74) | 36.2% (119) | 37.5% (162) | 33.9% (131) | 27.4% (92) | 30.2% (45) | 35.0% (55) | 35.5% (27) |
|  |  | **PERIPHERY** | 80.6% (319) | 52.6% (173) | 49.8% (215) | 61.7% (238) | 67.6% (227) | 65.8% (98) | 53.5% (84) | 53.9% (41) |
| BM-MSC | **TRANSCRIPTIONALLY ACTIVE** | **INTERIOR** | 26.5%  (26) | 40.2% (39) | 25.9% (43) | 34.7% (52) | 58.9% (103) | 63.2% (146) | 44.4% (88) | 70.9% (195) |
|  |  | **INTERMEDIATE** | 63.3% (62) | 51.5% (50) | 56.6% (94) | 52.7% (79) | 32.6% (57) | 28.6% (66) | 41.9% (83) | 25.5% (70) |
|  |  | **PERIPHERY** | 10.2%  (10) | 8.2% (8) | 17.5% (29) | 12.6% (19) | 8.6%  (15) | 8.2% (19) | 13.6% (27) | 3.6% (10) |
|  | **TRANSCRIPTIONALLY INACTIVE** | **INTERIOR** | 1.7% (6) | 2.9% (6) | 2.5% (7) | 3.0% (5) | 9.4% (12) | 8.7% (13) | 17.2% (23) | 19.5% (24) |
|  |  | **INTERMEDIATE** | 20.9% (73) | 35.7% (74) | 19.9% (55) | 25.6% (42) | 26.3% (34) | 33.6% (50) | 24.6% (33) | 42.3% (52) |
|  |  | **PERIPHERY** | 77.4% (271) | 61.4% (127) | 77.5% (214) | 71.3% (117) | 64.3% (83) | 57.7% (86) | 58.2% (78) | 38.2% (47) |

**Suppl. Table 4.** Nuclear distribution of transcriptionally active and inactive alleles within the nuclei with only one active allele. In total, 649 cells were analyzed in the AD-MSC system and 542 in BM-MSC.

|  |  | AD-MSC | BM-MSC |
| --- | --- | --- | --- |
|  |  | % of observed alleles  (number of alleles) | |
| TRANSCRIPTIONALLY ACTIVE | **INTERIOR** | 44.8% (291) | 44.3% (240) |
|  | **INTERMEDIATE** | 48.5% (315) | 46.9% (254) |
|  | **PERIPHERY** | 6.6% (43) | 9.0% (49) |
| TRANSCRIPTIONALLY INACTIVE | **INTERIOR** | 5.1% (33) | 6.8% (37) |
|  | **INTERMEDIATE** | 36.2% (235) | 31.9% (173) |
|  | **PERIPHERY** | 58.7% (381) | 61.1% (331) |
